# Associations of a multidimensional polygenic sleep health score and a sleep lifestyle index with disease outcomes and their interaction in a clinical biobank

**DOI:** 10.1016/j.sleh.2025.02.009

**Published:** 2025-04-12

**Authors:** Valentina Paz, Hannah Wilcox, Matthew Goodman, Heming Wang, Victoria Garfield, Richa Saxena, Hassan S. Dashti

**Affiliations:** a Instituto de Psicología Clínica, Facultad de Psicología, Universidad de la República, Montevideo, Uruguay; b Grupo Cronobiología, Universidad de la República, Montevideo, Uruguay; c MRC Unit for Lifelong Health and Ageing at UCL, Institute of Cardiovascular Sciences, Faculty of Population Health Sciences, University College London, London, United Kingdom; d Pharmacology and Therapeutics, Systems Molecular and Integrative Biology, Health & Life Sciences, University of Liverpool, Liverpool, United Kingdom; e Center for Genomic Medicine, Massachusetts General Hospital and Harvard Medical School, Boston, Massachusetts, United States; f Department of Anesthesia, Critical Care and Pain Medicine, Massachusetts General Hospital and Harvard Medical School, Boston, Massachusetts, United States; g Brigham and Women’s Hospital, Harvard Medical School, Boston, Massachusetts, United States; h Medical and Population Genetics, Broad Institute, Cambridge, Massachusetts, United States; i Division of Sleep Medicine, Harvard Medical School, Boston, Massachusetts, United States; j Division of Nutrition, Harvard Medical School, Boston, Massachusetts, United States

**Keywords:** Multidimensional sleep, Polygenic scores, Lifestyle behaviors, Phenome-wide association study, Clinical disorders, Mental health

## Abstract

**Objectives:**

Sleep is a complex behavior regulated by genetic and environmental factors impacting disease outcomes. However, the effect of multidimensional sleep encompassing several sleep dimensions on common diseases, specifically mental health disorders, has yet to be fully elucidated. Using the Mass General Brigham Biobank, we examined the association of multidimensional sleep with disease outcomes and investigated whether sleep behaviors modulate genetic predisposition to unfavorable sleep on mental health diseases.

**Methods:**

We generated a Polygenic Sleep Health Score using previously identified single nucleotide polymorphisms and constructed a Sleep Lifestyle Index based on self-reported questions and electronic health records; tested their association; performed phenome-wide association analyses between these indexes and clinical phenotypes; and analyzed their interaction on prevalent mental health diseases. A total of 15,884 participants were included in the analysis (mean age 54.4; 58.6% female).

**Results:**

The Polygenic Sleep Health Score was associated with the Sleep Lifestyle Index (β = 0.050, 95% CI = 0.032, 0.068) and with 114 disease outcomes spanning 12 disease groups, including obesity, sleep, and substance use disease outcomes (p < 3.3 × 10^−5^). The Sleep Lifestyle Index was associated with 458 disease outcomes spanning 17 groups, including sleep, mood, and anxiety disease outcomes (p < 5.1 × 10^−5^). A total of 108 disease outcomes were associated with both indexes, spanning 12 disease groups. No interactions were found between the indexes on mental health diseases.

**Conclusions:**

Favorable sleep behaviors and genetic predisposition to healthy sleep may independently protect against disease, underscoring the impact of multidimensional sleep on population health and the need for prevention strategies focused on healthy sleep habits.

## Introduction

Sleep disturbance has numerous adverse health consequences, including psychiatric and cardiometabolic disorders.^1^ The impact of sleep on disease outcomes has predominantly been studied by considering individual aspects of sleep, such as duration or timing separately. However, a shift in recent years has emphasized sleep as a multidimensional construct and operationalized sleep health as a composite measure encompassing several different aspects. Combining individual sleep characteristics effectively generates an index reflecting overall “good” or “bad” sleep.^2,3^ Several studies have used composite sleep metrics to investigate the relationship between sleep and different outcomes, particularly mental health outcomes, as psychiatric disorders are often associated with multifaceted sleep disturbances. These include studies reporting associations between “unhealthy” sleep scores, derived from self-report and actigraphy, and more depressive symptoms^4^ and higher risk of cardiovascular conditions,^5^ and between “healthy” sleep scores and better mental well-being^6^ and lower psychological distress.^7^

Sleep is a complex behavior regulated in part by environmental and genetic factors.^8,9^ Recent genome-wide association studies (GWAS) have identified genetic variants robustly associated with composite sleep,^10–12^ enabling the computation of Polygenic Risk Scores (PRS) (predictors of genetic susceptibility to traits or diseases) of multidimensional sleep.^13^ Combining PRS with phenotypic risk factors could help elucidate the interplay between genetic and environmental influences on disease risk.^14^ This inquiry is essential due to the large role of behavior and environmental constraints in sleep health in addition to sleep genetics.^15^ In particular, although mounting evidence links multidimensional sleep to mental health outcomes, whether sleep behaviors can modulate genetic predispositions remains poorly understood. Investigating this interaction is crucial for assessing the potential of altering sleep patterns to reduce the growing psychiatric burden worldwide.^16,17^

Large clinical biobanks combine electronic health records (EHR) with genetic data and health surveys, providing resources to systematically interrogate genetic and lifestyle factors that influence multidimensional sleep and their relationship with hundreds of clinical phenotypes through phenome-wide scans.^18–20^ In the present study, we leveraged the Mass General Brigham (MGB) Biobank to examine the association of multidimensional sleep (based on composite metrics of phenotypic and genetic data) with disease outcomes and to investigate whether sleep behaviors can modulate sleep-related genetic predisposition. To achieve this, we (1) calculated a Polygenic Sleep Health Score for each participant based on GWAS of a composite sleep health score, (2) constructed an index of “healthy” sleep based on data from self-reported questions regarding sleep patterns and EHR, and calculated a phenotypic Sleep Lifestyle Index for each participant, (3) tested the association between the genetic and phenotypic scores to validate the Polygenic Sleep Health Score in an independent population, (4) conducted a hypothesis-free phenome-wide association analysis (PheWAS) to identify disease outcomes associated with both scores, and (5) analyzed the interaction between the Polygenic Sleep Health Score and the Sleep Lifestyle Index on the “top hits” for mental health diseases from the PheWAS.

## Methods

### Sample

Study participants were patients from the MGB Biobank. The MGB Biobank is a healthcare enterprise clinical cohort from the MGB healthcare network in Massachusetts. The MGB Biobank links EHR with genetic and lifestyle data. Since 2009, patients have been recruited online through the patient portal or in-person across multiple MGB community-based primary care facilities and specialty tertiary care centers. The recruitment strategy has been described previously.^19^ Written informed consent was obtained from all patients upon enrollment (Spanish translation was available to promote patient inclusivity). The present study protocol was approved by the MGB Institutional Review Board (#2018P002276). At the time of the present analysis (03/2023), 140,915 patients had enrolled in the biobank.

### Polygenic Sleep Health Score

Among the enrolled patients, 64,639 patients (45.9% of the total) had provided blood samples available for genotyping. DNA from samples was genotyped using the Infinium Global Screening Array-24 version 2.0 (Illumina). Imputation was performed using the Michigan Imputation server with the Trans-Omics for Precision Medicine (TOPMed) (version r2) reference panel,^21^ and haplotype phasing was performed using Eagle version 2.3. As previously described, the genetic data were quality-controlled, excluding low-quality genetic markers and samples.^22^ Pairs of related individuals (kinship > 0.0625) were identified, and one sample from each related pair was excluded. Using TRACE and the Human Genome Diversity Project, principal components of ancestry were computed to correct for the population substructure.^23,24^ Participants of non-European ancestry (24.9% of the cohort) were excluded from the analysis to limit genetic heterogeneity in the present study.

Effect estimates for the Polygenic Sleep Health Score were derived from the most recent large-scale GWAS of composite sleep in UK Biobank participants of European ancestry aged 40–69 (n = 413,904). In this GWAS, a clinical additive sleep health score was derived from five favorable binary attributes of underlying ordinal sleep traits from self-report (7–8 hours of sleep duration, early chronotype, few insomnia symptoms, no snoring, and no excessive daytime sleepiness),^12^ following previous studies.^11,25^ For each participant in the present MGB Biobank study, a composite Polygenic Sleep Health Score was generated using Polygenic Risk Score-Continuous Shrinkage (PRS-CS).^26^ This method is based on Bayesian regression and places a continuous shrinkage prior on single nucleotide polymorphism effect sizes. The UK Biobank European ancestry linkage disequilibrium (LD) panel was used for LD pruning. A total of 239,446 single nucleotide polymorphisms were included in the score following clumping. The score was standardized with a mean of 0 and a standard deviation (SD) of 1.

### Sleep Lifestyle Index

All participants enrolled in the MGB Biobank were invited to complete an optional Health Information Questionnaire (HIQ) composed of lifestyle and family history questions. The following four questions related to sleep habits were asked with responses in half-hour increments: “In considering your longest sleep period, what time do you usually go to bed on weekdays or work or school days?” and “In considering your longest sleep period, what time do you usually wake up on weekdays or work or school days?” (both also asked for “weekends or days off”). At the time of analysis, 65,248 (46.30%) participants responded to the optional questionnaire. Self-reported bedtimes between 08:00 AM and 02:00 PM (weekday *n* = 329; weekend *n* = 613) and self-reported wake times between 06:00 PM and 12:00 AM (weekday *n* = 141; weekend *n* = 287), likely resulting from AM/PM misreporting, were set to missing. Improbable time in bed < 3 or > 18 hours (weekday *n* = 73; weekend *n* = 74) was set to missing, consistent with previous analyses.^27^

Using responses from these questions, the following was calculated: (1) time in bed as the weighted average weekly time in bed with 5/7 weighting for weekdays and 2/7 for weekends; (2) time in bed irregularity as the absolute value of the difference between weekday and weekend time in bed; (3) sleep midpoint as the midpoint of bed and wake times on weekends; and (4) social jetlag as the absolute difference in weekend and weekday sleep midpoint. From EHR data, sleep medications and sleep disorders, including insomnia and sleep apnea, based on the International Classification of Diseases (ICD)-9/−10 billing codes were determined. Participants with at least two codes for the same diagnosis on two separate dates within 5 years of completing the HIQ (to support cross-sectional analyses) were considered to have a sleep disorder, and those with no relevant code within this 5-year time window were considered free of sleep disorders (those with only one code were set to missing).

A Sleep Lifestyle Index was constructed based on similar attributes considered in the UK Biobank GWAS for a clinical additive sleep health score and other sleep indices based on available data.^5,28,29^ The index aggregated exposure to each of the following sleep behaviors: (1) adequate time in bed (≥7 hours and ≤9 hours per night); (2) regular time in bed (difference < 60 minutes between weekday and weekend time in bed); (3) healthy sleep midpoint (between 2:00 AM and 4:00 AM); (4) absence or mild/moderate social jetlag (< 2 hours); (5) not taking any medication known to affect sleep, such as those used to treat insomnia, anxiety, or circadian disorders (the list of medication is shown in Supplementary Material Table 1); (6) no recent diagnosis of any insomnia-related disorders; (7) no recent diagnosis of any sleep-related breathing disorders; and (8) no recent diagnosis of any other sleep disorder (the list of disorders is shown in Supplementary Material Table 2). Participants were assigned one point for each healthy sleep behavior.^3^ The Sleep Lifestyle Index scores ranged from 0–8, with higher scores reflecting more favorable (less problematic) sleep behaviors. Cross-trait correlations are presented in Supplementary Material Table 3. The associations between the index and each sleep behavior are presented in Supplementary Material Table 4.

In contrast to the Polygenic Sleep Health Score, the Sleep Lifestyle Index encompasses factors such as time in bed irregularity, social jetlag, use of medication, and sleep disorders (whereas the genetic index only considers symptoms of sleep disorders). Additionally, even though sleep midpoint, a proxy of chronotype, is part of our Sleep Lifestyle Index, the genetic index “chronotype” is based on a question regarding morningness-eveningness preferences. Lastly, our lifestyle index included “time in bed,” while the genetic index focuses on “sleep duration,” which does not account for time spent in bed without sleeping and includes naps.

### Clinical outcomes

The clinical phenotypes were determined from the ICD-9/−10 billing codes in the EHR.^19^ Codes were mapped to 1846 phenome-wide association study (PheWAS) codes (i.e., clinical phenotypes “phecodes”) based on clinical similarity. Same-day duplicated diagnoses and non-ICD-9/−10 codes were removed. Participants with at least two codes for a given disorder within 5 years of completing the HIQ were considered cases, and those with no relevant codes were considered controls.^20,30^

### Statistical analysis

We tested associations between the Polygenic Sleep Health Score and the Sleep Lifestyle Index, and between the Polygenic Sleep Health Score and each sleep attribute included in the Sleep Lifestyle Index using linear or logistic regression models adjusted for age, sex, genotyping array, batch, and principal components of ancestry (primary model) and further adjusted for employment, education, exercise, smoking, alcohol intake, body mass index, and Charlson Comorbidity Index^31^ (fully adjusted model). Although the indexes exhibit some overlap, we chose to analyze the association between them because they are distinct and constructed from different populations (UK Biobank and MGB Biobank). Analyzing the association between these distinct indexes allows for testing the replication of the Polygenic Sleep Health Score in an independent population, which ascertains sleep behaviors through different measures. The description of the covariates is shown in Supplementary Material Table 5.

We conducted a PheWAS for the Polygenic Sleep Health Score with 1354 disease outcomes (with at least 100 cases in the analytical sample) in 47,082 unrelated adult participants of European ancestry with high-quality genetic data using the PheWAS R package.^18^ We also conducted a PheWAS for the Sleep Lifestyle Index with 898 disease outcomes in 15,884 patients (33.7% of genotyped European patients) diagnosed within 5 years of completing the HIQ.

**Fig. 1.**
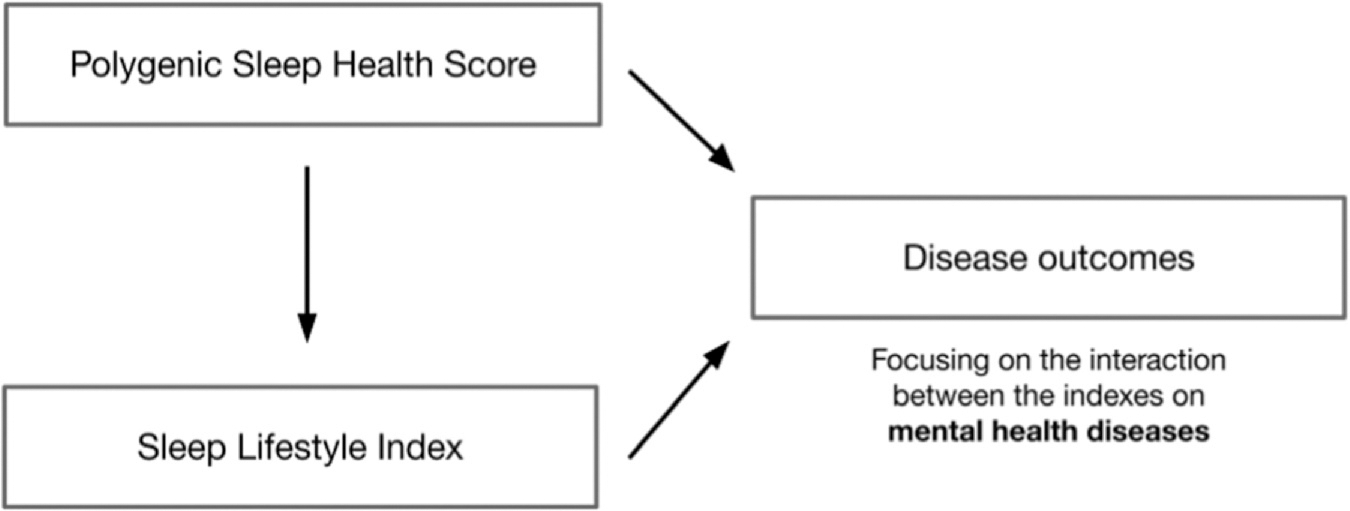
Associations explored in the study. The Polygenic Sleep Health Score was derived from a GWAS of composite sleep in UKB participants (based on five favorable sleep traits). The Sleep Lifestyle Index was constructed from a Health Information Questionnaire with questions regarding the sleep habits of MGB patients. GWAS, genome-wide association studies; MGB, Mass General Brigham; UKB, UK Biobank

We tested associations between the Polygenic Sleep Health Score and each disease outcome using logistic regressions adjusted for age, sex, genotyping array, batch, and principal components of ancestry. We tested associations between the Sleep Lifestyle Index and each disease outcome using logistic regressions adjusted for age, sex, employment, education, exercise, smoking, alcohol intake, body mass index, and the Charlson Comorbidity Index. Finally, we conducted interaction tests between the Polygenic Sleep Health Score and the Sleep Lifestyle Index for the top-five mental health diseases associated with both indexes in the PheWAS by further adding an interaction term between the indexes. Considering that our cohort is not powered to detect interaction effects across all possible outcomes with sufficient statistical rigor, the interaction analysis we conducted is exploratory in nature (see Fig. 1 for a description of the associations examined in the study).

Significance was determined at Bonferroni *P*-value cutoffs accounting for the total number of tests (e.g., for PheWAS results for the Sleep Lifestyle Index *P* value = .05/898 = 5 × 10^−5^). Descriptive statistics are presented as mean ± standard deviation. All analyses were performed using R statistical computing (version 2022.12.0; The R Foundation for Statistical Computing, Vienna, Austria).

## Results

**Fig. 2.**
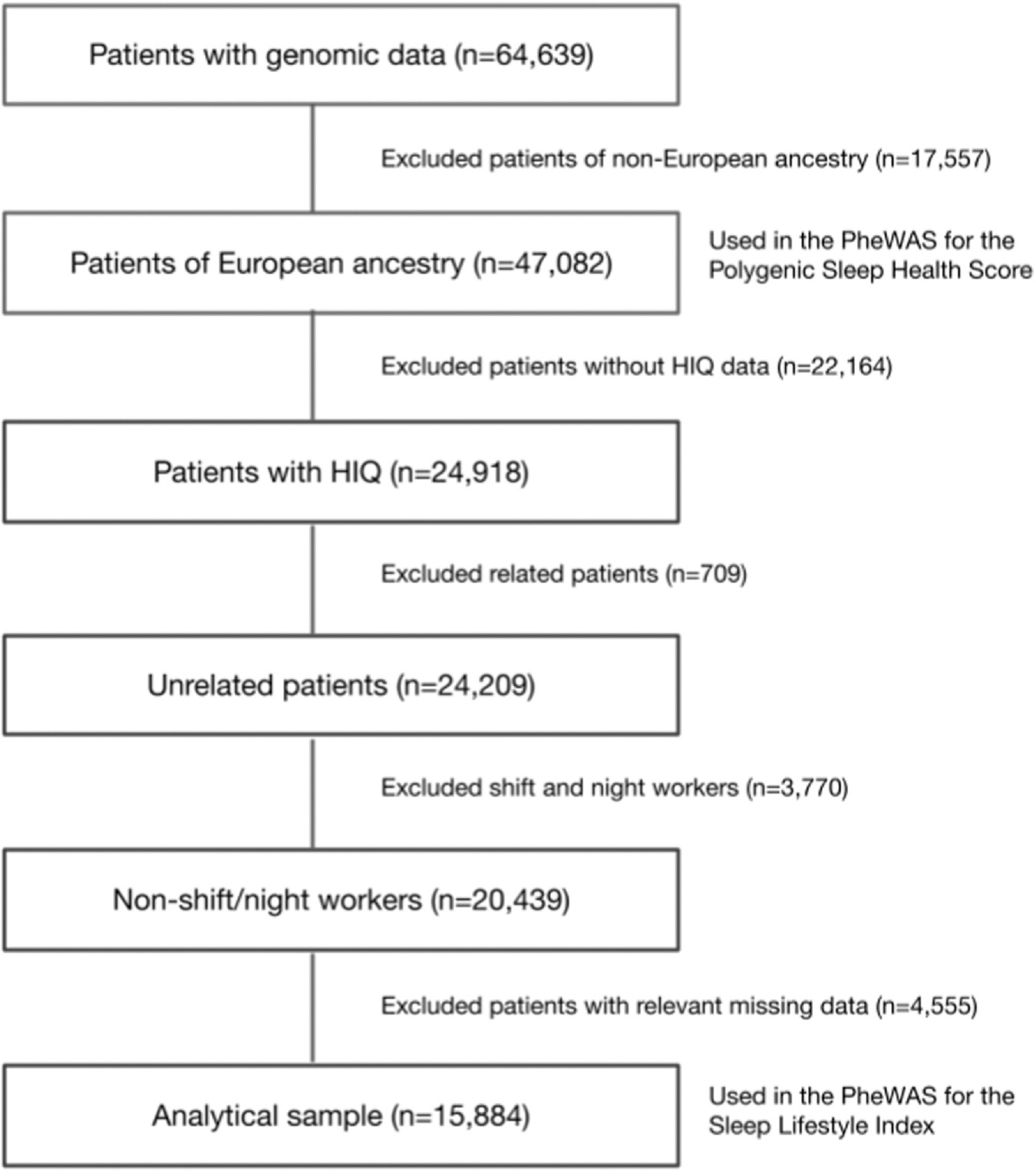
Flowchart of patients included in the analyses and exclusion criteria. HIQ, Health Information Questionnaire; PheWAS, phenome-wide association analysis

A total of 47,082 adult patients of European ancestry were included in the genetic analysis (mean age = 60.4 ± 17.0; 53.8% female), and 15,884 patients were included in the lifestyle analysis (mean age = 54.4 ± 16.3; 58.6% female) (Fig. 2).

**Fig. 3.**
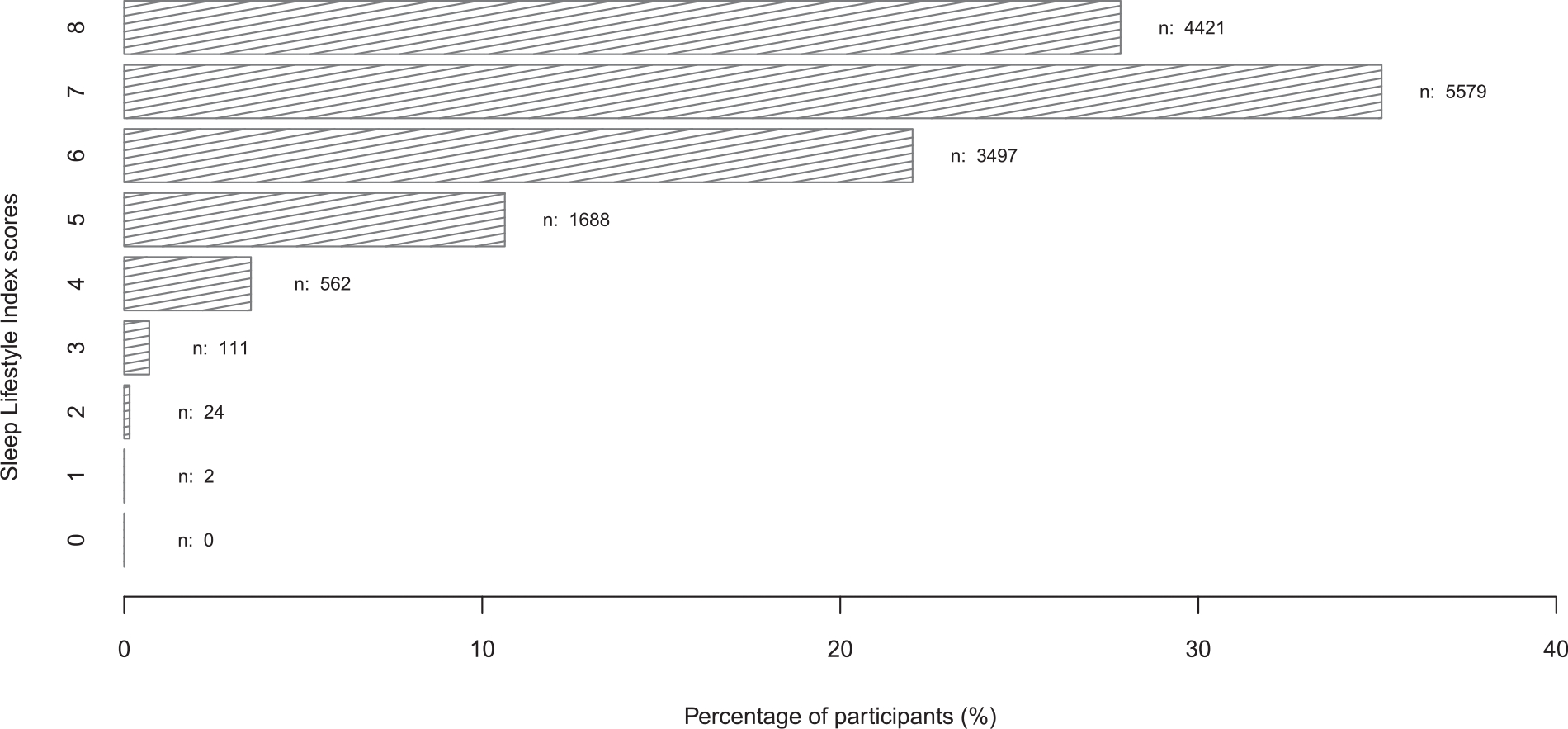
Histogram of Sleep Lifestyle Index scores based on self-reported sleep traits and data from electronic health records. The scores ranged from 0–8, with higher scores reflecting more favorable (less problematic) sleep behaviors

The cohort average for time in bed was 8.15 ± 1.13 hours, time in bed regularity was 0.78 ± 0.94 hours (range: 0–12 hours), sleep midpoint was 03:23 ± 01:41, and social jetlag was 0.86 ± 0.82 hours (range: 0–10.5 hours). The prevalence of insomnia-related disorders, sleep-related breathing disorders, and any other sleep disorders based on recent diagnoses were 1.9%, 3.5%, and 2.9%, respectively. All participants presented at least one of the healthy sleep lifestyle traits, while 4421 participants (27.83%) presented with all eight traits. Most participants presented with at least half of the healthy sleep behaviors ascertained (adequate time in bed, time in bed regularity, healthy sleep midpoint, and mild social jetlag) (Fig. 3).

The Polygenic Sleep Health Score was associated with the composite Sleep Lifestyle Index (primary model: β = 0.077, 95% CI = 0.058, 0.095; fully adjusted model: β = 0.050, 95% CI = 0.032, 0.068). Each SD increment in the Polygenic Sleep Health Score was associated with several components of the index, including less sleep irregularity (primary model: β = − 0.026, 95% CI = − 0.041, − 0.011; fully adjusted model: β = − 0.018, 95% CI = − 0.033, − 0.003), earlier sleep midpoint (primary model: β = − 0.066, 95% CI = − 0.093, − 0.039; fully adjusted model: β = − 0.057, 95% CI = − 0.084, − 0.030), less social jetlag (primary model: β = − 0.022, 95% CI = − 0.034, − 0.010; fully adjusted model: β = − 0.018, 95% CI = − 0.030, − 0.007), and, in the primary model only, higher odds of not having sleep-related breathing disorders (primary model: OR = 1.165, 95% CI = 1.065, 1.275; fully adjusted model: OR = 1.075, 95% CI = 0.980, 1.178) and higher odds of not having any other sleep disorder (primary model: OR = 1.165, 95% CI = 1.057, 1.285; fully adjusted model: OR = 1.082, 95% CI = 0.979, 1.196). The Polygenic Sleep Health Score was not associated with adequate time in bed, insomnia-related disorders, and medication (*P* value > .05).

### PheWAS findings

**Fig. 4.**
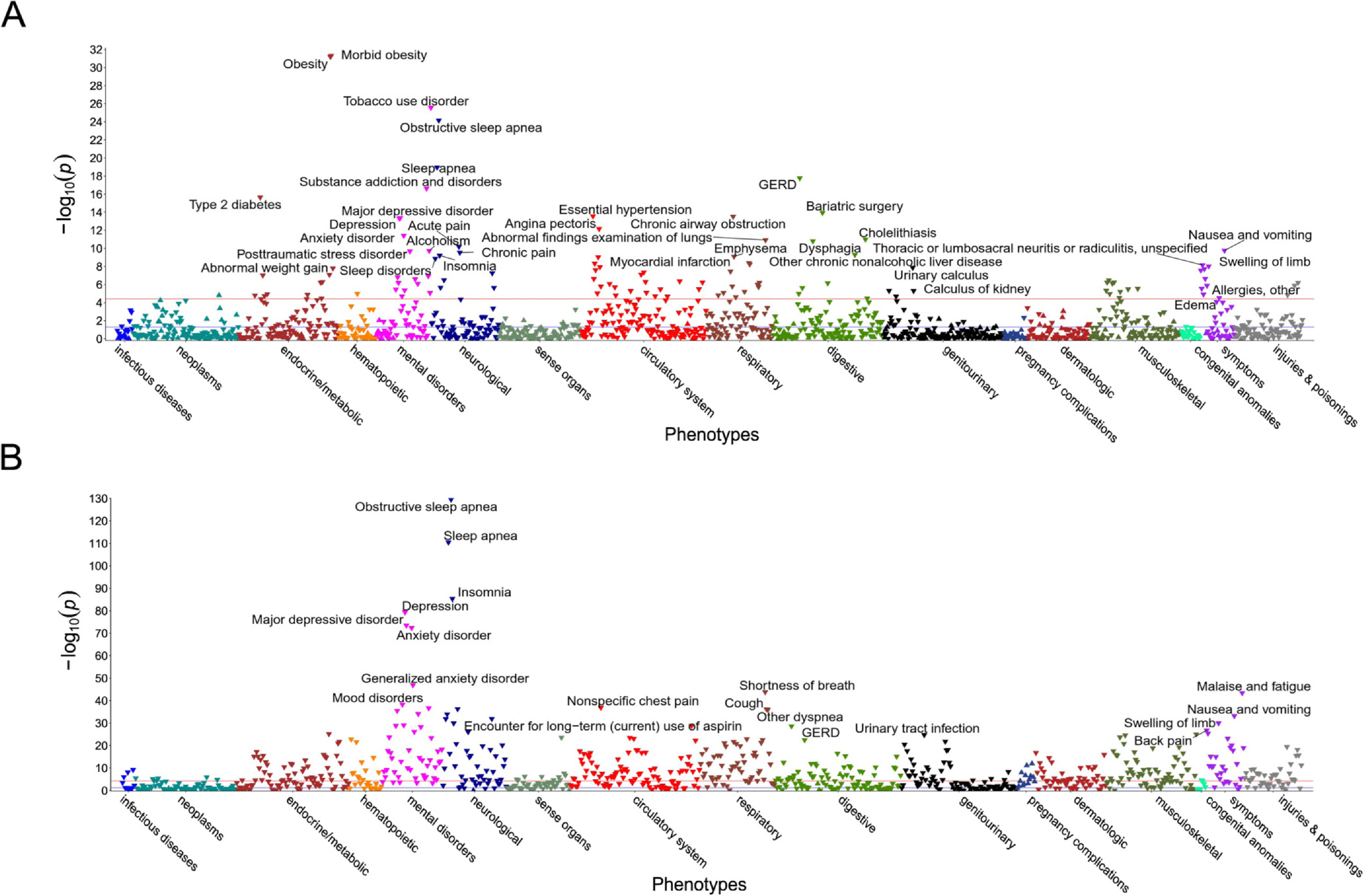
(A) Manhattan plot of phenome-wide associations between the Polygenic Sleep Health Score and disease outcomes (grouped by broad disease groups), in a model adjusting for age, sex, genotyping array, batch, and principal components of ancestry. (B) Manhattan plot of phenome-wide associations between the Sleep Lifestyle Index and disease outcomes (grouped by broad disease groups), in a model adjusting for age, sex, employment, education, exercise, smoking, alcohol intake, body mass index, and the Charlson Comorbidity Index. Notes: The −log_10_P value of the association is shown on the y-axis. The horizontal lines represent Bonferroni-corrected *P*-value cutoffs (the red line represents *P* value = 5 × 10^−5^; the blue line represents *P* value = 8 × 10^−4^). Upward triangles indicate positive associations, and downward triangles indicate negative associations

PheWAS results for the Polygenic Sleep Health Score are presented in Fig. 4A and in Supplementary Material Table 6. Significant findings were evident for 114 disease outcomes spanning 12 disease groups (p < 3.3 × 10^−5^). The strongest associations were Morbid obesity (OR = 0.822, 95% CI = 0.796, 0.850) and Obesity (OR = 0.864, 95% CI = 0.844, 0.886). The Polygenic Sleep Health Score was negatively associated with mental health diseases, accounting for 14.0% of all findings. The 10 strongest associations for mental health diseases were Tobacco use disorder (OR = 0.881, 95% CI = 0.861, 0.902), Substance addiction and disorders (OR = 0.858, 95% CI = 0.828, 0.889), Major depressive disorder (OR = 0.905, 95% CI = 0.882, 0.929), Depression (OR = 0.906, 95% CI = 0.883, 0.930), Anxiety disorder (OR = 0.916, 95% CI = 0.893, 0.939), Alcoholism (OR = 0.869, 95% CI = 0.833, 0.908), Post-traumatic stress disorder (OR = 0.848, 95% CI = 0.806, 0.892), Alcohol-related disorders (OR = 0.888, 95% CI = 0.850, 0.928), Mood disorders (OR = 0.904, 95% CI = 0.871, 0.939), and Adjustment reaction (OR = 0.924, 95% CI = 0.897, 0.952) (Fig. 6). The results were similar when adjusting for body mass index (BMI) (see Supplementary Material Table 7). We additionally conducted the PheWAS in the subset of participants who completed the HIQ as a sensitivity analysis. Significant associations were found only with Obesity (OR = 0.851, 95% CI = 0.798, 0.908), Essential Hypertension (OR = 0.892, 95% CI = 0.844, 0.943), and Eating Disorder (OR = 0.654, 95% CI = 0.531, 0.805). The effect estimates for Obesity and Essential Hypertension were similar to those observed in the larger sample. The association with Eating Disorder emerged only in the subset that completed the HIQ (see Supplementary Material Table 8). However, the results were weaker when adjusting for the Sleep Lifestyle Index (see Supplementary Material Table 9).

PheWAS results for the Sleep Lifestyle Index are presented in Fig. 4B and in Supplementary Material Table 10. Significant findings were evident for 458 disease outcomes spanning 17 disease groups (p < 5.1 × 10^−5^). The strongest associations were Obstructive sleep apnea (OR = 0.535, 95% CI = 0.508, 0.562) and Sleep apnea (OR = 0.466, 95% CI = 0.436, 0.498). Similarly, the Sleep Lifestyle Index was negatively associated with mental health diseases for 9.0% of significant findings. The 10 strongest associations for mental health diseases were Depression (OR = 0.668, 95% CI = 0.640, 0.696), Major depressive disorder (OR = 0.683, 95% CI = 0.655, 0.711), Anxiety disorder (OR = 0.693, 95% CI = 0.666, 0.721), Generalized anxiety disorder (OR = 0.662, 95% CI = 0.626, 0.700), Mood disorders (OR = 0.630, 95% CI = 0.588, 0.675), Adjustment reaction (OR = 0.719, 95% CI = 0.683, 0.756), Altered mental status (OR = 0.654, 95% CI = 0.612, 0.698), Dysthymic disorder (OR = 0.550, 95% CI = 0.500, 0.605), Bipolar (OR = 0.641, 95% CI = 0.593, 0.692), and Memory loss (OR = 0.676, 95% CI = 0.632, 0.724) (Fig. 6). The results were similar when adjusting for the Polygenic Sleep Health Score (see Supplementary Material Table 11).

**Fig. 5.**
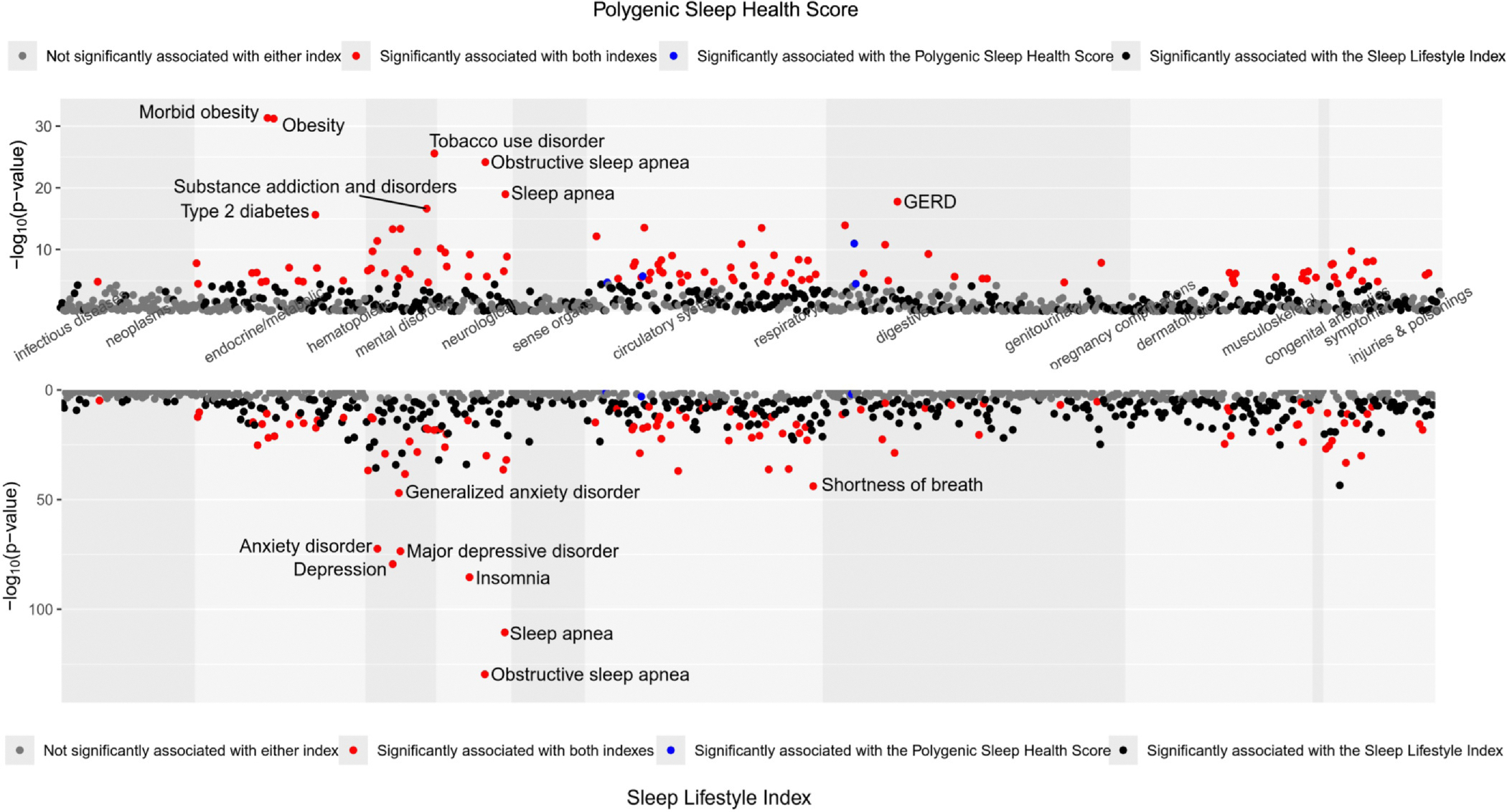
Miami plot of phenome-wide associations between the Polygenic Sleep Health Score and disease outcomes (top panel) and the Sleep Lifestyle Index and disease outcomes (bottom panel). The disease outcomes are grouped by broad disease groups. Gray dots indicate disease outcomes that are not significantly associated with either index. Red dots indicate disease outcomes that are significantly associated with both indexes. Blue dots indicate disease outcomes that are significantly associated with the Polygenic Sleep Health Score. Black dots indicate disease outcomes that are significantly associated with the Sleep Lifestyle Index. The disease outcomes with the name indicated are the top-eight outcomes associated with each index. Notes: The −log_10_P value of the association is shown on the y-axis

**Fig. 6.**
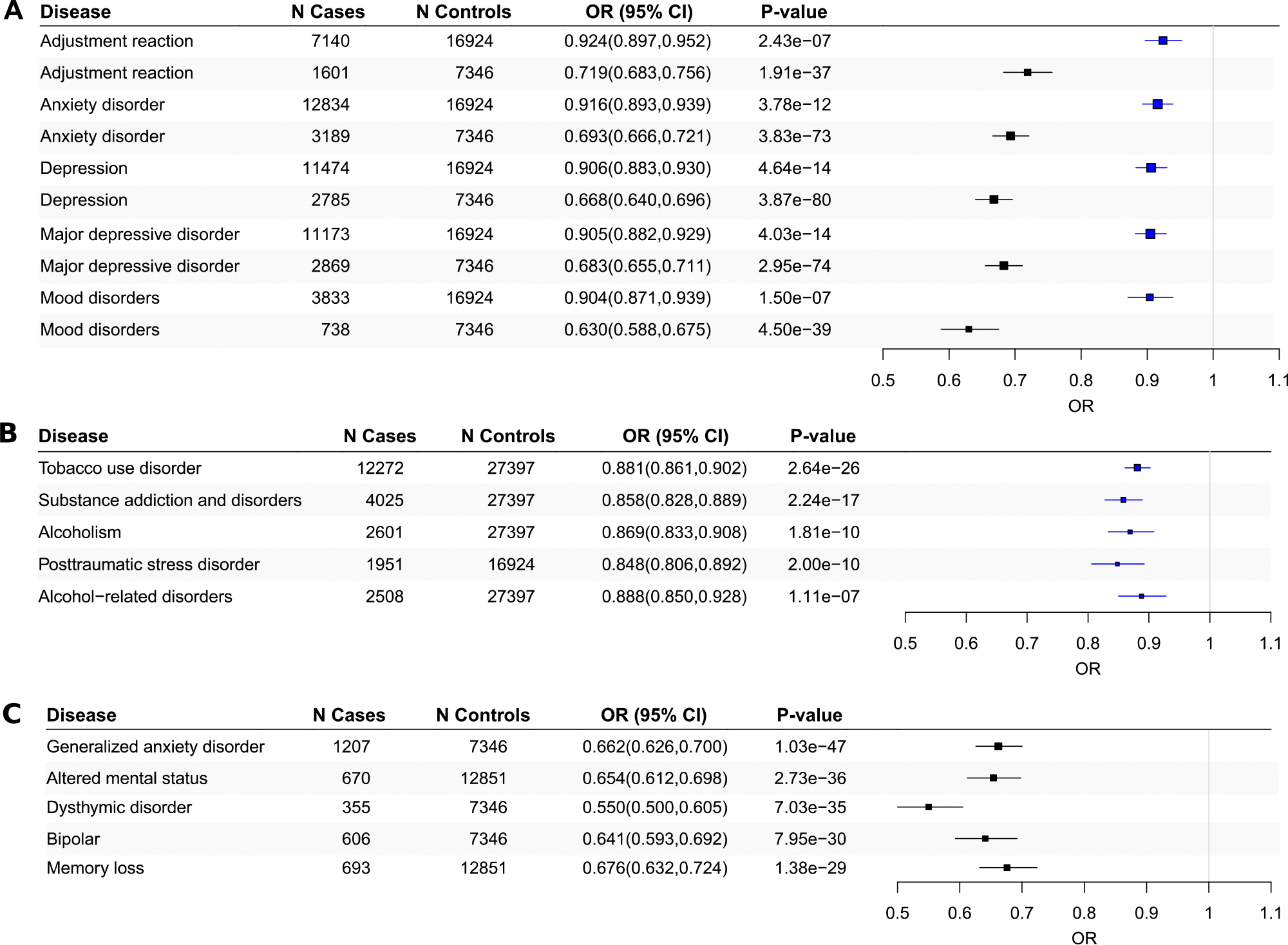
(A) Phenome-wide associations between the Polygenic Sleep Health Score (blue) and the Sleep Lifestyle Index (black) and the five mental health diseases strongly associated with both indexes. (B) Phenome-wide associations between the Polygenic Sleep Health Score and the other five mental health diseases strongly associated with the index. (C) Phenome-wide associations between the Sleep Lifestyle Index and the other five mental health diseases strongly associated with the index. OR, odds ratio; 95% CI, 95% confidence interval

A total of 108 disease outcomes were significantly associated with both indexes. These associations are highlighted in Fig. 5, with a complete list provided in Supplementary Material Table 12. Taking into account that five mental health outcomes were strongly associated with both indexes (Fig. 6A, see description in Supplementary Material 13) and previous work linking multidimensional sleep with mental health,^4,6,7^ we investigated whether these indexes interact to explain them. No significant interactions were observed between the indexes on these mental health diseases (all *P* > .05) (Table 1).

## Discussion

In the present study, we examined the link between genetic and behavioral multidimensional sleep indexes with disease outcomes and investigated whether sleep behaviors can modulate genetic predisposition. To achieve this, we calculated, for the first time, a polygenic score for composite sleep and constructed a lifestyle index based on sleep behaviors. Both indexes were associated with each other and with disease outcomes, including mental, neurological, and endocrine/metabolic disease outcomes. However, significant evidence of the specified parametric interactions between the indexes was not observed for the prevalent mental health conditions analyzed, suggesting the potential that improving sleep habits could help reduce disease risk, regardless of genetic predisposition.

We found that each standard deviation increment in the genetic index was associated with a 0.050–0.077 unit increase (0.625%–0.963%) in the lifestyle index. Although the effect estimates are modest, its cumulative effect over a lifetime may accrue more substantial influence. Previous studies have reported associations between polygenic scores for self-reported sleep duration, insomnia, and chronotype with their corresponding traits.^32,33^ Thus, our findings provide further support for the use of sleep polygenic scores as a complementary measure to self-reported sleep habits. We recognize, however, that validation in additional cohorts is necessary to ensure replicability and generalizability.

Moreover, we found that the genetic index was consistently negatively associated with disease outcomes; however, these associations weakened when the models were adjusted for the Sleep Lifestyle Index, suggesting that sleep habits may be a key mediating factor in understanding the relationship between genetic predisposition to healthy sleep and disease risk. Furthermore, when the PheWAS was conducted in the subset of participants who completed the HIQ, significant associations were found only with three disease outcomes, suggesting that PheWAS results for the Polygenic Sleep Health Score should be taken with caution. The lifestyle index was strongly associated with several diseases, which is consistent with the role of healthy sleep behaviors in improving overall health and the benefit of routine assessment of sleep disturbances in clinical services. In this line, two recent systematic reviews have reported that unhealthy sleep patterns, such as short and long sleep durations, high sleep variability, and late sleep timing, are associated with disease outcomes.^34,35^

Mental disorders were strongly associated with both indexes, particularly mood, anxiety, and reaction to severe stress and adjustment disorders. Specifically, the genetic index was associated with 8%−10% lower odds of having these disorders, while the lifestyle index was associated with 28%−37% lower odds. Noteworthily, these disorders are some of the most frequently diagnosed psychiatric conditions.^36^ The link between sleep difficulties and psychiatric illness is well-recognized; however, the effect of sleep on mental health is not fully understood. Current evidence suggests a causal role of sleep behaviors in developing these disorders. For example, Mendelian randomization (MR) studies have found an increased risk of anxiety in those with insomnia,^37^ and bidirectional associations between insomnia and depression.^38^ Further, a recent study has reported substantial polygenic overlap between sleep-related traits and some mental conditions, including depression.^39^ Moreover, the GWAS used to generate the genetic index reported genetic correlations between the composite sleep health score and several mental health conditions, including mental distress, anxiety, and depression.^12^ In addition, a meta-analysis of randomized controlled trials found that improving sleep quality reduces depression and anxiety symptoms.^40^ Nonetheless, some MR studies did not find causal effects between chronotype or insomnia on depression.^41,42^ This knowledge emphasizes the importance of thoroughly investigating the connection between sleep and mental health, offering new possibilities for therapeutic interventions.

Metabolic diseases were also associated with the indexes. Particularly, obesity was the phenotype most strongly associated with the genetic index. The link between sleep and obesity has been extensively reported.^43^ Previous meta-analyses have revealed that sleep durations outside the normal range increase the incidence of obesity.^44^ In agreement, prior research has reported U-shaped positive genetic correlations between short and long sleep durations and BMI, waist circumference, and waist-to-hip ratio.^27^ However, it remains elusive whether the association between sleep duration and BMI is causal.^45^ Sleep breathing disorders are also highly associated with obesity,^46^ and a robust causal effect of insomnia on higher BMI has been reported in an MR study.^47^ Regarding genetic risk, a previous study on the MGB Biobank indicated that a polygenic index for sleep duration was associated with obesity.^32^

Finally, we did not find statistical interactions of genetic and lifestyle sleep factors on the five most prevalent mental health diseases associated with both indexes. Recent work has shown that short/long sleep duration modifies genetic risk for adverse lipid profiles^48^ and blood pressure.^49^ Similarly, some gene-sleep interaction studies suggest favorable sleep behaviors may attenuate genetic predisposition to obesity.^43^ However, for other phenotypes, such as type 2 diabetes, little compelling data support gene-lifestyle interactions.^50^ Thus, one possibility for our null findings is that, as for diabetes, lifestyle factors independently predict the mental health diseases studied. It is also possible that we have inaccurate or incomplete patient diagnoses, which would contribute to the misclassification of cases. Nevertheless, the absence of evidence for the tested statistical interactions may suggest that improving sleep behaviors may ameliorate disease risk, regardless of genetic background.

Our study has several strengths. First, unique to the MGB Biobank is the linking of large and diverse medical data enriched for disease with genetic and sleep information generally unavailable in other clinical biobanks. Second, using composite sleep metrics has several advantages, including the recognition that sleep dimensions do not occur in isolation and the possibility of analyzing gradients of healthy sleep beyond the absence/presence of sleep disturbances.^51^ Third, a score derived from genetic markers should act independently of confounders that could influence the associations between sleep and disease outcomes. Finally, while composite metrics may strengthen predictive power, they can also obscure the distinct contribution of individual sleep traits.^2^

Limitations of the study should also be acknowledged. The study was restricted to participants of European ancestry, as the discovery GWAS was conducted in Europeans^12^; future work in diverse racial and ethnic groups is necessary for the generalization of findings and to promote health equity. Furthermore, these results should be replicated in diverse age groups and nonclinical cohorts to confirm generalizability. We focused on the MGB biobank; thus, diagnosis data are likely incomplete as we did not consider information from other medical facilities. Validating these indexes with diseases from additional electronic medical records is necessary. The low prevalence of insomnia-related disorders and sleep-related breathing disorders could be reflecting the underdiagnosis of these conditions in the EHR.^52^ The exclusion of rare diseases (i.e., < 100 cases) in the PheWAS analyses due to small case numbers warrants future exploration of multidimensional sleep effects on these disease outcomes. Furthermore, our analyses were based on self-reported data. The modest HIQ response rate introduces potential selection bias of older, more women, more White, and less healthy participants as previously noted,^29^ while the single-survey administration limits insights into behavior stability over time. This study examined interactions between the indexes using additive models for mental health conditions only; nonparametric interactions were not tested. Future studies should further investigate these interactions using nonparametric models and explore interactions for additional disorders. Given the exploratory nature of the PheWAS analysis, our findings should be interpreted as preliminary and as providing a basis for future hypothesis-driven studies that will require further validation. Lastly, considering the cross-sectional nature of our study, along with potential genetic pleiotropy, we cannot infer the direction of causality. Reverse causality remains a possibility, as pre-existing conditions may lead to changes in sleep behaviors, potentially biasing the observed relationships.

## Conclusions

The present study explored how genetic susceptibility to healthy sleep and beneficial sleep habits were associated with various disease outcomes within a clinical cohort. While genetic and phenotypic sleep factors were linked to several diseases, no interactions were evident between these factors on mental health diseases. Overall, our findings emphasize the relevance of sleep for a healthy life, demonstrate the pleiotropic nature of sleep genetics, and underscore the importance of leveraging clinical biobanks in advancing precision medicine research. Further research is needed on the association between multidimensional sleep and disease outcomes in diverse clinical settings.

## Supplementary Material

1

## Figures and Tables

**Table 1:** Interactions between the Polygenic Sleep Health Score and the Sleep Lifestyle Index on disease outcomes

	Primary model		Fully adjusted model	
Disease	OR (95% CI)	*P* value	OR (95% CI)	*P* value

Adjustment reaction	0.984 (0.935, 1.036)	.523	0.983 (0.934, 1.035)	.501
Anxiety disorder	0.992 (0.954, 1.032)	.704	0.988 (0.948, 1.030)	.558
Depression	1.004 (0.963, 1.046)	.863	1.002 (0.960, 1.046)	.918
Major depressive disorder	0.996 (0.956, 1.038)	.843	0.994 (0.954, 1.036)	.795
Mood disorders	0.986 (0.921, 1.056)	.696	0.975 (0.909, 1.047)	.491

Abbreviations: OR, odd ratio; 95% CI, 95% confidence interval.

Disease outcomes were limited to the five mental health diseases strongly associated with both indexes.

## Data Availability

Data are available from the Mass General Brigham Human Research Office/Institutional Review Board at Mass General Brigham for researchers who meet the criteria for access to confidential data.

## Appendix A. Supporting information

Supplementary data associated with this article can be found in the online version at doi:10.1016/j.sleh.2025.02.009.
